# ‘’Myth Busting in Infectious Diseases’’: A Comprehensive Review

**DOI:** 10.7759/cureus.57238

**Published:** 2024-03-30

**Authors:** Ali Almajid, Shatha Almuyidi, Shatha Alahmadi, Sarah Bohaligah, Layal Alfaqih, Abdulelah Alotaibi, Albatul Almarzooq, Asmaa Alsarihi, ⁠Zaina Alrawi, Rahaf Althaqfan, Rahma Alamoudi, Sultan Albaqami, Alaa H Alali

**Affiliations:** 1 Internal Medicine, King Fahad Specialist Hospital, Dammam, SAU; 2 Medicine, Umm Al-Qura University, Makkah, SAU; 3 Medicine, Imam Abdulrahman Bin Faisal University, Dammam, SAU; 4 Medicine, Taif University, Taif, SAU; 5 Medicine, Vision College, Riyadh, SAU; 6 Applied Medical Sciences, Taibah University, AlMadinah, SAU; 7 Medicine, King Abdulaziz University, Jeddah, SAU; 8 Applied Medical Sciences, King Khalid University, Khamis Mushait, SAU; 9 Medicine, Ibn Sina National College for Medical Studies, Jeddah, SAU; 10 Internal Medicine, Al Noor Specialist Hospital, Makkah, SAU; 11 Infectious Diseases, King Saud Medical City, Riyadh, SAU

**Keywords:** cefazolin, streptococcus pyogenes, trimethoprim-sulfamethoxazole, urinary tract infections, adverse effects, misconceptions, myths, antibiotic resistance, antibiotics

## Abstract

Antibiotics have played a pivotal role in modern medicine, drastically reducing mortality rates associated with bacterial infections. Despite their significant contributions, the emergence of antibiotic resistance has become a formidable challenge, necessitating a re-evaluation of antibiotic use practices. The widespread belief in clinical practice that bactericidal antibiotics are inherently superior to bacteriostatic ones lacks consistent support from evidence in randomized controlled trials (RCTs). With the latest evidence, certain infections have demonstrated equal or even superior efficacy with bacteriostatic agents. Furthermore, within clinical practice, there is a tendency to indiscriminately order urine cultures for febrile patients, even in cases where alternative etiologies might be present. Consequently, upon obtaining a positive urine culture result, patients often receive antimicrobial prescriptions despite the absence of clinical indications warranting such treatment. Furthermore, it is a prevailing notion among physicians that extended durations of antibiotic therapy confer potential benefits and mitigate the emergence of antimicrobial resistance. Contrary to this belief, empirical evidence refutes such assertions. This article aims to address common myths and misconceptions within the field of infectious diseases.

## Introduction and background

Antibiotics have revolutionized modern medicine, saving countless lives by combating bacterial infections. Several decades ago, managing even minor infections posed a significant challenge and often resulted in fatal outcomes. Despite the substantial progress made since the introduction of antibiotics, bacterial infections have once again become a critical concern, primarily due to the rise of antibiotic resistance [1]. However, alongside their remarkable efficacy, numerous myths and misconceptions have emerged surrounding their use. One example is that the majority of patients are prescribed antibiotics upon hospital discharge for infections, even in cases where there is no clinical necessity; the antibiotic's spectrum of activity is incomplete, or the dosage is inappropriate [2-5]. 

Despite decades of experience, the clinical optimization of antibiotics continues to be a subject of ongoing exploration. A conventional belief in medicine asserts that bactericidal agents are more effective than bacteriostatic ones. Intuitively, it may be assumed that bacteriostatic antibiotics merely halt bacterial growth, while bactericidal agents actively kill or eliminate bacteria. The logical inclination is often to favor the latter, assuming their superior efficacy in eradicating bacterial infections [6]. Considering the abundance of misconceptions within the domain of infectious diseases, the aim of this article is to elucidate the myths surrounding infectious diseases, encompassing various branches within the field in light of current evidence.

## Review

Bactericidal versus bacteriostatic

In the medical community, there is a prevalent belief that bactericidal antibiotics exhibit greater potency compared to bacteriostatic counterparts. This assertion is grounded in the understanding that "cidal" agents eradicate bacteria, while "static" agents merely inhibit bacterial growth. As a result, there is a tendency to prioritize the prescription of bactericidal agents based on the perception of their enhanced efficacy in bacterial eradication. Nevertheless, it is essential to recognize that these widely held assumptions regarding the definitions of bacteriostatic and bactericidal may not entirely align with scientific principles [6]. All antibiotics categorized as bacteriostatic are capable of eliminating bacteria in laboratory conditions, albeit at concentrations that exceed their minimal inhibitory concentration (MIC) to a greater extent compared to bactericidal agents. Two important definitions need clarification. First, the MIC is the amount of a substance needed to stop visible bacterial growth after 24 hours in specific conditions like temperature and carbon dioxide level. Second, the minimum bactericidal concentration (MBC) is the amount of a drug needed to reduce bacterial density by 1,000 times after 24 hours in the same conditions. A bactericidal antibiotic has an MBC-to-MIC ratio of 4 or less, while a bacteriostatic agent has a ratio greater than 4 [6]. In clinical settings, the comparison between bactericidal and bacteriostatic antibiotics has been extensively explored through numerous RCTs across various infections. Surprisingly, these investigations have failed to establish a clinically significant distinction between the two categories. In fact, in certain infections, such as skin and soft tissue infections (SSTIs), in the comparison between 'linezolid/static versus vancomycin/cidal', bacteriostatic agents have demonstrated even greater efficacy than their bactericidal counterparts [7].

A meta-analysis involving 56 RCTs revealed that bactericidal agents are not inherently more effective than bacteriostatic agents [8]. The majority of trials conducted across various infections, such as SSTIs, nosocomial pneumonia, community-acquired pneumonia (CAP), aspiration pneumonia, typhoid fever, complicated intra-abdominal infections, Gram-positive bacteremia, genital chlamydia trachomatis infection, and bacterial vaginosis, showed no significant difference in efficacy between bacteriostatic and bactericidal agents. Out of the seven trials that identified a statistically significant difference in clinical outcomes, six trials indicated that bacteriostatic agents exhibited superior efficacy. These trials specifically involved linezolid/static versus ceftriaxone/cefpodoxime in pneumococcal pneumonia, linezolid/static versus vancomycin in pneumonia and SSTIs. The only trial that favored a bactericidal agent (imipenem) employed a suboptimal dosage of the bacteriostatic agent (tigecycline). Therefore, RCTs do not provide evidence supporting the superiority of bactericidal agents in clinical settings [8]. However, no RCTs were conducted to assess the comparison between 'cidal' and 'static' agents in the context of meningitis or endocarditis. Therefore, the meta-analysis results mentioned earlier may not be applicable to meningitis and endocarditis.

Duration of antibiotics therapy

In the hospital setting, where there is significant demand for antibiotics, shortening the duration of antibiotic therapy emerges as a critical strategy to mitigate unnecessary antibiotic utilization. By reducing hospital stays, mitigating the emergence of antibacterial resistance, minimizing drug-related adverse effects, and preventing superinfections such as fungal and *Clostridium difficile* infections, the practice of administering antibiotics for shorter duration proves pivotal in optimizing patient care and antibiotic stewardship efforts [9,10].

The traditional duration of antibiotic therapy is typically based on the notion that a week consists of seven days, as established by Roman Emperor Constantine the Great almost two thousand years ago [11]. Consequently, many standard antibiotic regimens span from 7 to 14 days. However, relying solely on this ancient definition seems inadequate for guiding contemporary medical practices [11].

Regarding efficacy, lots of studies conducted to prove short-term antibiotic regimens has demonstrated comparable efficacy to their long-term counterparts. Unfortunately, the persistent belief in long treatments became even stronger because of another unfounded idea that prematurely ending such treatments increases resistance [12]. However, resistance is significantly more prone to develop with prolonged antibiotic courses [13,14].

Fortunately, in recent years, more than 120 randomized controlled trials (RCTs) have compared the efficacy of shorter versus traditional, longer courses of antibiotic treatment [15]. These studies have found that shorter courses are non-inferior for various conditions such as community-acquired and nosocomial pneumonia, acute exacerbation of chronic bronchitis and sinusitis, complicated urinary and intra-abdominal infections, Gram-negative (GN) bacteremia, acute bacterial skin infections, osteomyelitis, septic arthritis, and even neutropenic fever [16-19]. It's important to note that the shorter treatment regimens studied did not typically adhere to the traditional seven-day week as defined by Constantine. Furthermore, a comprehensive meta-analysis led by Kasparian et al., which compared short-term with long-term durations in acute cholangitis, encompassing 1,313 patients in the analysis, revealed no significant disparities in mortality rates between antibiotic regimens lasting two-three days and those of longer duration [20]. Moreover, recurrence rates and hospitalization duration exhibited no variation across all study cohorts. Consequently, the findings imply that both short- and long-course antibiotic treatments might yield comparable efficacy in managing mortality and recurrence rates associated with acute cholangitis in adult patients. Table 1 presents a summary of meta-analyses of clinical trials examining common infections, comparing short-term versus long-term antibiotic duration.

**Table 1:** A summary of meta-analyses of clinical trials comparing short-term versus long-term antibiotic duration in common infections CAP: Community-acquired pneumonia; RCTs: Randomized-controlled trials; CTs: Clinical trials; VAP: Ventilator-associated pneumonia; AECOPD: Acute exacerbation of chronic obstructive pulmonary disease; GN: Gram-negative; IPD: Individual participant data

Condition	Study	Cohort	Duration	Result	Reference
CAP	Meta-analysis of 4 RCTs	Pediatrics	3-5 days vs. 7-10 days	Equally effective	[21]
CAP	Meta-analysis of 21 CTs	Adults	≤ 6 days vs. ≥ 7 days	Equally effective, lower mortality with short-term	[22]
VAP	Meta-analysis of 5 RCTs	Adults	≤ 8 days vs. ≥ 10-15 days	Equally effective	[23]
AECOPD	Bayesian meta-analysis of 22 RCTs	Adults	Super-short: 1-3 days; Short: 4-6 days; Standard: 7-9 days; Long: ≥ 10 days	Equally effective, 4-6 days might be the safest	[24]
GN Bacteraemia (Enterobacterals)	Meta-analysis of IPD involving 3 RCTs	Adults	≤ 7 days vs. > 7 days (7-14)	Equally effective	[25]
Meningitis	Meta-analysis of 6 RCTs	Pediatrics	≤ 7 days vs. 10 days or double the days of equivalent short-term course	Equally effective	[26]

Oral versus intravenous administration of antimicrobials

IV antibiotics are often recommended for patients admitted to the hospital to rapidly achieve peak concentration, as IV antibiotics have higher bioavailability compared to oral antibiotics. However, prescribing them routinely upon hospital admission isn't warranted. Factors to consider when deciding on the administration route include the oral agent's bioavailability, the patient's ability to swallow or absorb medications orally, illness severity, clinical stability, identified pathogen, and infection site [27]. While oral administration is recognized as a cost-effective method, a notable obstacle arises from the perception that oral medications might not attain comparable therapeutic levels to IV counterparts. However, the expanding accessibility of newer and more efficacious oral agents has enhanced acceptance towards this transition approach [27].

 Utilizing oral antibiotics offers several advantages, such as facilitating readiness for discharge, decreasing the likelihood of IV catheter infections and related complications, lowering costs, and reducing workload. In contrast to previous clinical practice, contemporary evidence indicates that certain severe infections, including infective endocarditis, osteomyelitis, and bacteremia arising from urinary tract infection (UTI), exhibit favorable responses to treatment through the transition to a highly bioavailable oral medication [28,29-35]. Additionally, a major clinical trial, the "OVIVA trial," demonstrated that oral therapy was noninferior to IV therapy during the first six weeks, showing comparable rates of treatment success at one year in adults with bone or joint infections [35].

A Cochrane review was conducted to assess the effectiveness of oral versus IV antibiotics in febrile neutropenic patients. It concluded that oral therapy can serve as a suitable alternative to IV antibiotic treatment in patients with cancer and febrile neutropenia (excluding those with acute leukemia) who demonstrate hemodynamic stability, do not have organ failure, and are not affected by pneumonia, central line infection, or severe SSTI [36].

Cefazolin for central nervous system (CNS) infections 

Cefazolin is a first-generation cephalosporin antibiotic known for its broad-spectrum antibacterial activity, especially against Streptococcal and Staphylococcal species. Historically, cefazolin, as a first-generation cephalosporin antibiotic, has been pivotal in managing CNS infections due to its effective pharmacokinetics and broad antibacterial activity [37]. However, a common misconception about cefazolin is the belief that it is inappropriate for treating bacterial meningitis. The reported negative outcomes in case series involving patients receiving cephalothin for invasive streptococcal infections suggested that these occurrences were due to poor penetration of cephalothin across the blood-brain barrier. Although there are no documented breakthrough meningitis cases associated with cefazolin, its use as a therapeutic agent for CNS infections has likely been avoided due to its classification alongside other first-generation cephalosporins, such as cephalothin [38].

A case report has indicated that continuous infusion of cefazolin could achieve therapeutic levels and sterilize cerebrospinal fluid (CSF), potentially aiding in bacterial eradication [39]. The report, which involved a patient with methicillin-sensitive *Staphylococcus aureus* (MSSA) ventriculitis, demonstrated that administering at least 8 grams of cefazolin daily via continuous infusion led to CSF sterilization within 48 hours of initiating cefazolin treatment [39].

A retrospective study was conducted to assess the efficacy of cefazolin in treating MSSA-related meningitis by investigating its penetration into the meninges [40]. Among the 17 patients included, 47% received cefazolin treatment, primarily administered via continuous infusion. The median CSF concentration of cefazolin was 2.8 mg/L. The results of this study indicate that cefazolin can attain therapeutic concentrations in CSF, thereby suggesting its potential as a viable treatment option for MSSA meningitis [40]. Furthermore, a prospective pharmacokinetic study was conducted to assess the distribution of cefazolin into the CSF in critically ill adults with neurological injuries. Blood and CSF samples were collected from 15 patients receiving cefazolin intravenously for external ventricular drain (EVD) prophylaxis. The study revealed median CSF concentrations of 2.97 mg/L for peak levels and 1.59 mg/L for trough levels, suggesting viable therapeutic options for meningitis or ventriculitis caused by MSSA. Further investigation through clinical trials is warranted to confirm these findings [41].

Antibiotics adverse effects and contraindications

Doxycycline and Children

Antibiotics are implicated in tooth discoloration across pediatric populations. Historically, tetracycline-class antibiotics have been extensively employed to combat various infections in children [42,43]. During this time frame, clinicians frequently noted the occurrence of yellow pigmentation on the teeth of young patients attending follow-up clinics, an observation that correlates with earlier findings linking skeletal pigmentation in children to tetracycline exposure [44]. Historical concerns regarding tooth discoloration in children under eight years old are associated with older tetracycline-class medications, which exhibit a greater propensity to bind with calcium compared to more contemporary counterparts like doxycycline [45].

In 2013, Todd et al. conducted a blinded study to assess tooth discoloration following the administration of doxycycline to children under eight years old in a community with high rate of Rocky Mountain spotted fever. The study involved 58 children under eight years old who received doxycycline for an average duration of 7.3 days, with an average dosage of 2.3 mg/kg, compared to 213 children who never received doxycycline. The study found no significant differences in tooth discoloration between children who received doxycycline and those who did not [46]. Additionally, the American Academy of Pediatrics Red Book indicates that doxycycline can be safely administered for up to 21 days regardless of age [47].

Linezolid and Serotonin Syndrome

Over the past 15 years, serotonin toxicity, also known as serotonin syndrome, has become a more prevalent and significant clinical concern in the field of medicine. This coincides with the introduction of numerous new antidepressants capable of increasing serotonin (5-HT) levels in the CNS [48]. The oxazolidinones share structural similarities with toloxatone, a recognized inhibitor of monoamine oxidase (MAO) [49]. Linezolid, a synthetic oxazolidinone antibiotic, exhibits mild and reversible inhibition of both MAO-A and MAO-B [50,51]. In humans, MAO exists in two forms: types A and B. These enzymes play a crucial role in metabolizing monoamine neurotransmitters such as epinephrine, norepinephrine, serotonin, and dopamine. MAO-A primarily metabolizes epinephrine, norepinephrine, and serotonin. The simultaneous use of a nonselective MAO inhibitor (e.g., phenelzine) and a selective serotonin reuptake inhibitor (SSRI) is well-documented to induce serotonin syndrome [49,52]. Nevertheless, real-world data from a retrospective study involving 1,743 patients determined that the incidence of serotonin syndrome among individuals administered both linezolid and a serotonergic agent was 0.06%, as assessed by the Sternbach criteria, and recorded as 0% based on the Hunter criteria [53]. Furthermore, in another retrospective study spanning a six-year period and involving 1,134 patients prescribed linezolid, 215 patients were concurrently taking antidepressants, and the incidence of serotonin syndrome in the entire cohort was less than 0.5% [54].

Nitrofurantoin and Creatinine Clearance

Nitrofurantoin is an antibiotic that is mainly used as a first-line agent in the treatment of uncomplicated cystitis [55]. Nonetheless, is use is limited in patients with creatinine clearance (CrCl) below 60 mL/min. Instances of adverse events associated with nitrofurantoin in patients with renal insufficiency primarily arise from treatments that exceed the recommended 5-day duration advised by the Infectious Diseases Society of America (IDSA) [55-57]. The product information for Macrodantin in 1988 specified a CrCl cutoff level of 40 mL/min, whereas the updated contraindication of less than 60 mL/min is stated in the 2003 Macrobid product information. The decision to avoid using nitrofurantoin in patients with a CrCl below 60 mL/min appears to stem from studies conducted in the 1950s and 1960s that investigated the urinary excretion of this drug in patients with varying degrees of kidney function [58]. These studies were criticized for their small sample sizes, lack of statistical rigor, and inconsistent methodologies, including unclear definitions of CrCl or renal impairment, as well as varied doses and treatment durations. Most data were gathered after a single dose of nitrofurantoin, using outdated formulations with lower bioavailability. However, they failed to establish a clear link between CrCl values and urinary nitrofurantoin concentrations or clinical outcomes. Importantly, they did not measure urinary concentrations but rather reported the quantities of nitrofurantoin in urine samples [58].

In contrast, three retrospective studies have investigated the use of nitrofurantoin in patients with impaired renal function and supported the safety and efficacy of nitrofurantoin in renal impairment. One study from 2009, involving hospitalized Canadian patients, found similar rates of clinical cure between those with CrCl >50 mL/min and those with CrCl ≤50 mL/min [59]. Adverse events were rare and comparable between the groups. Another study in 2013, focusing on outpatient Danish women, found no significant difference in overall treatment ineffectiveness between nitrofurantoin and trimethoprim (TMP), though adverse reactions were more common with nitrofurantoin, particularly in patients with impaired renal function (median CrCl = 38 mL/min) [60]. Similarly, a retrospective review of Canadian women aged 65 or older found that while a second antibiotic was more frequently prescribed for the nitrofurantoin group in patients with impaired renal function (median CrCl = 38 mL/min), there was no increased risk of treatment failure compared to those with median CrCl of 69 mL/min [61]. Moreover, The 2015 Beers Criteria for Potentially Inappropriate Medication Use in Older Adults provides a revised suggestion regarding the utilization of nitrofurantoin in individuals with compromised kidney function. The revised criteria reduced the renal function threshold to CrCl <30 mL/min [62]. Thus, the contraindication of avoiding nitrofurantoin in patients with a CrCl <60 mL/min may not be warranted.

Urinary tract infections

UTI is a frequently encountered infection; nonetheless, physicians often order urine cultures for patients without clear indications, resulting in the inappropriate prescription of antibiotics [63]. According to the Centers for Disease Control and Prevention (CDC), hospitals could potentially avoid 40% of the inappropriately prescribed antibiotics [64]. Individuals with asymptomatic bacteriuria (ASB) in the intensive care unit (ICU) and elderly patients experiencing confusion are more likely to receive antimicrobial treatment. However, the use of antibiotics for those with ASB, lacking clear indications, significantly contributes to the development of antibiotic resistance [63]. Factors such as urine color, smell, and the presence of pyuria (or positive leukocyte esterase) or bacteriuria should not be used in isolation for diagnosis [63-66]. Contamination should be suspected if the urine sample contains > 5 squamous epithelial cells, necessitating proper collection procedures [67]. Notably, not all bacteria found in catheter-obtained urine samples should be treated as a UTI; 89% of chronically catheterized patients exhibit bacteriuria [68]. Furthermore, there is no conclusive evidence indicating that patients with ASB will develop UTI or long-term complications like pyelonephritis, sepsis, hypertension, or renal failure in the future [69]. Elderly individuals presenting with confusion and falls do not always have UTI; alternative causes for altered mental status should be considered [69]. Lastly, the presence of candida in urine samples, particularly in catheterized patients, does not necessarily indicate a candida UTI or the need for antimicrobial prescriptions unless patients are at high risk, such as those on immunosuppressants or transplant recipients [70]. Therefore, doctors should integrate clinical symptoms and investigative findings to prevent over-treatment and unnecessary antimicrobial prescriptions.

Sterile pyuria is characterized by a concentration of ten or more white blood cells (WBC) per cubic millimeter in a urine sample. Research has demonstrated that 13.9% of women and 2.6% of men show a positive WBC count in their urine samples. It is crucial to note that not all occurrences of sterile pyuria signify an infection; a range of potential factors can lead to an elevated WBC count in a urine sample. These factors encompass sexually transmitted diseases, genitourinary tuberculosis, fungal infections, and various inflammatory and autoimmune conditions [71-77]. Furthermore, inflammation beyond the urinary tract and various urological conditions, such as pneumonia, urinary stones, radiation cystitis, and polycystic disease, can also present with sterile pyuria [78]. It is imperative to conduct a thorough assessment to identify the precise cause of sterile pyuria, guiding the formulation of an effective management plan. This strategy aids in reducing the improper and erroneous use of antimicrobials, ensuring that the treatment aligns with the underlying condition.

Screening and treating ASB is recommended for pregnant women and individuals undergoing invasive urologic procedures [79]. However, there are strong recommendations against screening and treating healthy women, older individuals, children, men, or those with diabetes, non-renal organ transplants, indwelling catheters, patients with urologic device implantation, or spinal cord injuries. Moreover, patients undergoing non-urological procedures do not need antibiotics for UTI, but they should receive standard antibiotics as prophylaxis 30-60 minutes before surgery [79]. The potential benefits of screening and treating ASB in these groups do not outweigh the associated risks. Additionally, the evidence for screening or treating patients with a post-kidney transplant beyond one month or those who are neutropenic was considered insufficient by the latest IDSA guidelines [79].

The efficacy of trimethoprim/sulfamethoxazole against *Streptococcus pyogenes*

Several in vitro studies have highlighted the unreliable activity of trimethoprim/sulfamethoxazole (TMP-SMX) against *Streptococcus pyogenes *(*S. pyogenes*), underscoring instances of resistance to TMP-SMX [80,81]. The findings of these in vitro studies are linked to high thymidine content, which is recognized for inhibiting TMP-SMX activity, in contrast to Mueller Hinton broth, which has low levels of thymidine [82]. This initial encounter led to the perception that TMP-SMX is not effective against *S. pyogenes*, resulting in its discouragement in clinical practice for many years. Furthermore, the latest clinical practice guidelines by the IDSA do not recommend the use of TMP-SMX in the initial empiric management of SSTI for coverage of *S. pyogenes* [83]. In contrast, a recent investigation involving 49 isolates of *S. pyogenes* revealed that all isolates exhibited susceptibility to TMP-SMX when assessed using the gold standard method of broth microdilution (BMD) [84]. Several other in vitro studies have provided supportive data on the activity of TMP-SMX against *S. pyogenes* [85-87]. The majority of clinical trials on TMP-SMX have concentrated on SSTI, where *Staphylococcus aureus* (*S. aureus*) is the primary pathogen [84,88-94]. Nevertheless, recent trials addressing impetigo and cellulitis unequivocally indicate that TMP-SMX is non-inferior to the standard of care in treating those diseases [95,96]. A trial conducted by Bowen et al. compared short-course oral co-trimoxazole with standard treatment using intramuscular benzathine benzylpenicillin in 508 patients from a pediatric population aged 3 months to 13 years with purulent or crusted non-bullous impetigo. Participants were randomly assigned to receive either benzathine benzylpenicillin or co-trimoxazole. The results demonstrated the non-inferiority of co-trimoxazole compared to benzathine benzylpenicillin in treating non-bullous impetigo, which is commonly caused by *S. aureus* and *S. pyogenes* [95]. In another trial involving 524 patients diagnosed with SSTIs, of which 280 (53.4%) presented with cellulitis, participants were randomly allocated to receive either clindamycin or TMP-SMX for a duration of 10 days. The study revealed no statistically significant variance in clinical cure rates between the clindamycin and TMP-SMX cohorts, irrespective of age or infection type [96].

Antimicrobial resistance

One of the most pivotal advancements in medical treatment has been the introduction of antibiotics, heralded as a potent form of chemotherapy. Sir Alexander Fleming's revelation of penicillin in 1928 marked the beginning of the antibiotic era [97,98]. Initially hailed for their remarkable efficacy in combating harmful bacteria, early optimism suggested that infectious diseases might eventually be eradicated. However, in recent decades, the emergence and proliferation of antibiotic-resistant pathogens, notably multidrug-resistant bacteria, have underscored a critical challenge [99]. This phenomenon highlights a fundamental misunderstanding of the intricate ecological and evolutionary dynamics within microbial ecosystems [99]. It is now evident that microbial communities exhibit extensive metabolic diversity, enabling them to deploy various defense mechanisms against selective pressures from their environment and human interventions such as antibiotics [100].

Combination Therapy Is Always the Best Approach

Utilizing more than one antimicrobial agent to hinder the development of resistance is not beneficial in all cases. This approach originates from early observations that employing multiple antimicrobial agents proved effective in preventing resistance emergence in *M. tuberculosis*. However, applying this strategy to bacteria with moderately complex or complex resistance poses challenges [101]. The prevalence of multidrug resistance in moderately complex bacteria and the emergence of such resistance during therapy in highly complex bacteria suggest that increasing the number of antibiotics may lead to an escalation in resistance. Examples such as cephalosporin selection of vancomycin resistance in *Enterococcus faecium* and fluoroquinolone selection for imipenem resistance in *Pseudomonas aeruginosa* (*P. aeruginosa*) illustrate instances of co-selection [102,103].

Resistance to Any Antibiotic Can Arise in Any Species

The reality is that resistance patterns vary based on the organism and antibiotic. Certain bacterial species, including *P. aeruginosa*, *Providencia stuartii*, and *S. marcescens*, are notable for their diverse resistance capabilities, indicating specificity to particular organisms. These species possess inherent resistance features, which are further enhanced by their ability to adapt genetically, allowing them to acquire and maintain resistance elements from various sources. Likewise, challenges similar to those posed by *P. aeruginosa*, *Providencia stuartii*, and *S. marcescens* are encountered with *S. haemolyticus* and enterococci among Gram-positive species [104,105]. On the other hand, certain species appear to encounter challenges in developing resistance. Non-faecal streptococci and clostridia have only shown resistance to a restricted range of antibiotics thus far [105]. Antibiotic selectivity. Rapid emergence of resistance to particular antibiotics like streptomycin and rifampicin. Specifically, short-term rifampicin monotherapy in non-tuberculous conditions has not consistently led to resistance [106]. On the opposite spectrum, there exist a handful of antibiotics that seldom encounter resistance. Nitrofurantoin and the polyene antifungal agents serve as prime examples of such cases [105].

Acquired Resistance Arises Solely Following Exposure to an Antibiotic

Contrary to common belief, resistance to an antimicrobial agent can occur even without prior exposure of the resistant organisms to the specific antibiotic in question. For example, an in vitro study conducted after the introduction of flucytosine revealed that a significant percentage of Candida albicans and Candida glabrata strains were resistant to flucytosine despite never having been exposed to the antibiotic [107]. Likewise, Enterococcus casseliflavus carries an intrinsic resistance to vancomycin [108]. Table 2 summarises the myths addressed in the current review.

**Table 2:** Summary of the myths addressed in the present review CNS: Central nervous system; MSSA: Methicillin-sensitive *Staphylococcus aureus*; CSF: Cerebrospinal fluid; CrCl: Creatinine clearance; TMP-SMX: Trimethoprim-sulfamethoxazole

Myth	True/Findings in the Study
Bactericidal antibiotics are inherently more effective than bacteriostatic ones	Bactericidal and bacteriostatic antibiotics show comparable efficacy in clinical settings, with some bacteriostatic agents demonstrating even greater efficacy than bactericidal ones
Longer courses of antibiotics are always better for treating infections	Shorter antibiotic courses are as effective as longer ones for various infections, reducing hospital stays, adverse effects, and costs without increasing resistance
Oral antibiotics are less effective than intravenous antibiotics	Oral antibiotics are equally effective as intravenous ones for certain infections, promoting earlier discharge, reducing catheter infections, and lowering costs
Cefazolin is inappropriate for treating bacterial meningitis	Cefazolin may be effective for treating CNS infections caused by MSSA, achieving excellent therapeutic levels in CSF
Doxycycline causes tooth discoloration in children below 8 years of age	Doxycycline does not cause tooth discoloration in children under 8 years old and can be safely administered for up to 21 days regardless of age
Linezolid and serotonin syndrome	Despite structural similarities to serotonergic drugs, linezolid has a low incidence of serotonin syndrome when used concurrently with serotonergic agents
Nitrofurantoin is contraindicated if CrCl is below 60 mL/min	Nitrofurantoin can be used safely in patients with CrCl above 30 mL/min, contrary to previous contraindications
TMP-SMX is ineffective against *Streptococcus pyogenes*	TMP-SMX is effective against *Streptococcus pyogenes*, showing non-inferiority to standard treatments for impetigo and cellulitis
Antibiotic resistance	Antibiotic resistance can occur without prior antibiotic exposure

## Conclusions

In conclusion, the examination of myths and misconceptions surrounding antibiotic use reveals critical insights for modern clinical practice. By addressing prevalent misconceptions, we can optimize patient care and stewardship efforts while combating the rising threat of antimicrobial resistance. To begin with, the distinction between bactericidal and bacteriostatic antibiotics lacks significant clinical relevance. Evidence suggests comparable efficacy across various infections, challenging the longstanding preference for bactericidal agents. Additionally, shorter antibiotic courses have emerged as non-inferior alternatives to longer regimens, challenging historical practices and offering potential benefits in mitigating resistance. Furthermore, we have addressed certain misconceptions surrounding specific antibiotics such as the belief that doxycycline should not be used in children under 8 years old, concerns about linezolid and serotonin syndrome, misconceptions regarding the contraindications of nitrofurantoin use when CrCl is below 60 mL/min, and explored the potential of cefazolin to achieve excellent concentrations in CSF, suggesting its potential usefulness in treating CNS infections. In essence, embracing evidence-based practices and dispelling entrenched myths are crucial steps towards optimizing antibiotic use. Safeguarding patient well-being and preserving antibiotic efficacy for future generations necessitate aligning clinical practice with current evidence.
